# Research Progress of Titanium-Based Alloys for Medical Devices

**DOI:** 10.3390/biomedicines11112997

**Published:** 2023-11-08

**Authors:** Madalina Simona Baltatu, Petrica Vizureanu, Andrei Victor Sandu, Carmen Solcan, Luminița Diana Hritcu, Mihaela Claudia Spataru

**Affiliations:** 1 Faculty of Materials Science and Engineering, “Gheorghe Asachi” Technical University of Iasi, 41 “D. Mangeron” Street, 700050 Iasi, Romania; cercel.msimona@yahoo.com (M.S.B.); sav@tuiasi.ro (A.V.S.); 2 Technical Sciences Academy of Romania, Dacia Blvd 26, 030167 Bucharest, Romania; 3 Romanian Inventors Forum, Str. Sf. P. Movila 3, 700089 Iasi, Romania; 4 Academy of Romanian Scientists, 54 Splaiul Independentei, 050094 Bucharest, Romania; 5 National Institute for Research and Development in Environmental Protection, 294 Splaiul Independentei, 060031 Bucharest, Romania; 6 Department of Public Health, Faculty of Veterinary Medicine, Iasi University of Life Sciences, Mihail Sadoveanu Street, No 3, 700490 Iasi, Romania; lumidih@yahoo.com (L.D.H.); mspatarufmv@yahoo.com (M.C.S.)

**Keywords:** titanium alloys, non-toxic elements, characterization

## Abstract

Biomaterials are currently a unique class of materials that are essential to improving the standard of human life and extending it. In the assent of the appearance of biomaterials that contain non-toxic elements, in this study, we examine a system of Ti25Mo7Zr15Ta*x*Si (*x* = 0, 0.5, 0.75, 1 wt.%) for future medical applications. The alloys were developed in a vacuum electric arc furnace and then studied from a structural, mechanical and in vivo assessment (on rabbits) perspective. The effect of the silicon addition was clearly seen in both the structural and the mechanical characteristics, standing out as beta alloys with a dendritic structure and lowering the mechanical properties as a result of the silicon addition. In experimental rabbits, the proliferation of mesenchymal stem cells was observed in the periosteum and peri-implant area, differentiating into osteoblasts and then into osteocytes. Osteoclasts were discovered within the cartilaginous islands that provide structural support to newly formed bone, playing a primary role in bone remodeling. The newly formed spongy tissue adhered to the fibrous capsule that surrounds the alloy, ensuring good osseointegration of metallic implants. The overexpression of Osteopontin, Metalloproteinase-2 (also known as gelatinase A), and Metallopeptidase-9 (also known as gelatinase B) underscores the processes of osteogenesis, bone mineralization, and normal bone remodeling.

## 1. Introduction

From the perspective of protecting health, biomaterials can be defined as “materials that possess new properties that make them suitable to come into immediate contact with living tissue without causing an immune rejection or an adverse reaction” [1,2,3].

Scientists can now model the properties of materials at high levels thanks to the knowledge they have accumulated over the previous 70 years. In order to meet the numerous service requirements of today’s complex society, tens of thousands of different materials have been developed with specific properties [4,5].

In reparative medicine, a broad diversity of biomaterials are used. Currently, commercially pure titanium (C.P.-Ti), Ti6Al4V, Ti6Al7Nb, CoCr and stainless-steel alloys [6,7,8] are used to fabricate orthopedic implants. Unfortunately, these materials have demonstrated a tendency to degrade over time due to various factors. They have a higher Young’s modulus than bone, low wear and corrosion resistance, and they are not biocompatible. It’s worth noting that understanding the properties of alloying elements can provide valuable insights into their suitability for biomedical applications. In this context, Young’s modulus (or elastic modulus) represents the measure of stiffness in a material. Alloying elements, such as Tantalum (Ta), Zirconium (Zr) and Silicon (Si), have specific values for Young’s modulus that play a pivotal role in determining their mechanical behavior. For instance, Tantalum possesses Young’s modulus close to that of bone, making it an attractive option for orthopedic implants. Zirconium, likewise, exhibits a favorable Young’s modulus, and when alloyed with other elements, it can improve wear and corrosion resistance. Silicon, often added in trace amounts, can influence the mechanical properties of the resulting alloy, including its Young’s modulus [8,9,10].

The identification and testing of the effectiveness of new types of implants are the essential stages for the design and development of the medical device industry. The most commonly utilized materials in medical applications are still titanium and its alloys because of their exceptional characteristics. They are mostly utilized in the domains of orthopedics, dentistry and cardiovascular medicine for the replacement of hard tissues because of their high tensile strength, satisfactory biocompatibility and good resistance to corrosion or wear [7,8,9,10]. Some studies [8] show that C.P. Ti, Ti-Nb-Zr and Ti-Mo alloys show superior corrosion resistance. While certain in vitro studies have demonstrated the superiority of Ti-based alloys over commercially pure titanium (C.P. Ti), others have indicated that Ta-*x*Zr alloys may enhance the adhesion, proliferation, and differentiation of osteogenic cells. In in vivo studies, alloys with a higher percentage of Ta have shown increased osteogenic activity, with titanium alloys containing Ta-30Zr emerging as a promising alternative [11]. However, Ta-Zr alloys show low elastic moduli and some biomechanical particularities, which are similar to those of human bone [12,13,14,15,16].

Tantalum is considered to be non-toxic and of medium biocompatibility [17]. In vitro studies have presented the proliferation and adhesion of osteoblasts to the surface of the porous tantalum rod showing a variety of shapes and intercellular connections [18]. In in vivo studies, researchers have described peri-implant vascular proliferation and the formation of new bone tissue and bone trabeculae directly connected to the alloy [12,19,20,21]. In a study on rats, Jugdaohsingh et al. [22] showed that silicon was found in bones as well as elastane and collagen of the connective tissues in organs such as the aorta, bone, trachea or tendon up to 50 fold compared with the non-connective viscera (liver, kidney and spleen), and even if the concentration of silicon had increased with age, the highest content was found in the connective tissue of young weanling rats.

Silicon (Si), which can be found as silicide and solid solution, is a crucial element in titanium (Ti) alloys. Some studies have demonstrated that the addition of silicon to titanium alloys can improve strength, creep resistance and oxidation resistance, as well as reduce fluidity, particularly at ambient temperature [22,23,24,25,26].

The biocompatibility of implant materials is the determining characteristic for the osseointegration process and implicitly for implant stability, reliability and durability. The concept of intrinsic biocompatibility is defined as “the ability of a material to induce an appropriate host response, in the case of a specific application”. In implantology, the concept of biocompatibility of materials must be combined with that of the bifunctionality of the implant, which implies the in vivo functional performance of the implant [27,28,29,30].

It is crucial to design materials with superior biocompatibility and high durability. Although a variety of materials are now used as biomaterials, titanium alloys are quickly replacing them as the most in-demand material for most applications [31,32,33,34,35,36,37,38,39].

**Figure 1:**
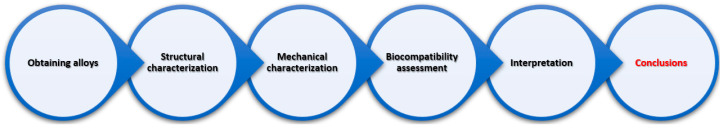
Scientific protocol flow overview.

This scientific study explores the microstructural and biological properties of a new Ti25Mo7Zr15Ta*x*Si (*x* = 0, 0.5, 0.75, 1 wt.%) system developed for medical applications. This study includes microstructure analyses, mechanical testing, and in vivo investigation of the chemical and biological characteristics of the alloys to evaluate their performance. Figure 1 provides an overview of the contents of this study and the methods used to achieve the research objectives.

## 2. Materials and Methods

### 2.1. Material Development

The production of titanium alloys is a complex process that poses several challenges, including the need for protective conditions and the high cost of metallurgical equipment. Titanium has a strong tendency to react with oxygen, hydrogen and nitrogen, which can cause gas dissolution in the melted metal during alloy production. To address these issues, specialized metallurgical equipment is required to enable processing under vacuum or a controlled argon atmosphere, which provides the necessary protection against gas contamination [40].

To ensure biocompatibility, high-purity chemical elements (99.5% for each element purchased from Alfa Aesar, Thermo Fisher Scientific, Waltham, MA, USA) were used to produce Ti-25Mo-7Zr-15Ta-xSi alloys. Upon analyzing the Ti-Mo binary diagram, it was observed that there is a broad range of potential chemical compositions that can provide excellent chemical stability for the alloy. The alloys obtained were Ti25Mo7Zr15Ta, Ti25Mo7Zr15Ta0.5Si, Ti25Mo7Zr15Ta0.75Si and Ti25Mo7Zr15Ta1Si. The effect of silicon on the alloys was analyzed by keeping the content of other alloying elements constant and by increasing the silicon content (*x*Si = 0.5; 0.75; 1%).

To obtain our Ti-based alloys for this study, vacuum arc remelting equipment was used. The device used for obtaining these alloys is widely employed [41]. Several steps are involved, including cutting and weighing the raw materials, charging them onto a copper plate, creating a vacuum, and finally, evacuating the semi-finished products.

### 2.2. Structural Characterization Methods

To emphasize the presence of pure chemical elements, we used a scanning electron microscope (SEM; Vega 2 LSH Tescan, Brno, Czech Republic).

The metal samples obtained from the Ti-Mo-Zr-Ta-Si system were examined using metallographic analysis. For the purpose of analyzing optical microstructures, it is recommended to utilize a Zeiss Axio Imager A1 upright microscope, which is equipped with multiple objectives. This microscope is manufactured by Carl-Zeiss-Strasse located in Oberkochen, Germany. In order to examine the metallographic structure of the samples, they were first polished and then subjected to etching with a reactive solution composed of 10 mL HF, 5 mL HNO_3_ and 85 mL H_2_O, for a duration of 30 s.

The identification of phases was conducted using Panalytical X’Pert Pro MPD equipment, manufactured by Malvern Panalytical Ltd. in the Malvern, UK. The data obtained from the equipment were processed using the Highscore Plus program.

### 2.3. Mechanical Properties

In mechanical engineering, indentation hardness tests are employed to determine a material’s resistance to deformation. Various tests are available where the material is indented until an impression is formed, either on a macroscopic or microscopic level.

The properties of the TiMoZrTaSi alloys obtained were evaluated through indentation using a Tribometer UMT 2 apparatus equipped with a Rockwell diamond penetrator (Bruker, Campbell, CA, USA). Instrumented indentation involves applying a sharp tip to the material’s surface and recording a force–displacement curve. These results provide valuable insights into the material’s mechanical behavior, such as hardness, elastic moduli, and plastic deformation. An essential aspect of instrumented indentation testing is the precise control of the tip’s force or displacement, which can be measured continuously throughout the indentation process. Advanced technology now allows accurate force control within a broad range.

### 2.4. In Vivo Biocompatibility Assessment

Interaction between the alloys and bone tissues was observed after surgical implantation into the tibial compacta of mature rabbits (*Oryctolagus cuniculus*) aged 8–10 months. A control rabbit received a stainless-steel orthopedic rod. Experimental protocol followed the regulations of the European and national legislation on the matter of experimentation on animals and ISO 10993-1:2018 procedures [42]. Additionally, prophylaxis, animal maintenance, intra- and post-surgery maneuvers and euthanasia of animals in the final stages of the experiment for taking biological samples were approved by the professional ethics committee of Faculty of Veterinary Medicine from Iasi [43,44]. To secure the alloy on the cranial tibial crest of anesthetized animals, we made a 0.5 cm incision and inserted an orthopedic rod, approximately 0.3 mm wide and 0.4–0.8 mm deep, into the control rabbit and implants into four experimental rabbits. Once the implants were fixed, we sutured the gaps, periosteum, regional fascia, and skin. To observe the position of the implants and recovery of the surrounding tissues, X-ray examinations were performed using an Intermedical Basic 4006 device (Grassobbio, Italy). The images were recorded using the X-CR Smart Examion digital system, with the images saved in DICOM format and read using the Radiant program. The experiment lasted 60 days, and CT scan evaluation was performed using General Electric LightSpeed 16 (Boston, MA, USA). The Hounsfield Unit (UH) scale was used to highlight the radiodensity of the alloys, implanted bones and surrounding structures.

*Histologic analysis.* After fixing in 10% formalin for 24 h, slices of implanted bone were decalcified over 6 weeks in 15% EDTA, with a pH of 7. The stems were dehydrated in decreasing ethanol concentrations, clarified with xylene, embedded in paraffin, and cut into 5 μm sections using a microtome. Using the standard hematoxylin–eosin protocol, 10 microscope slides from each paraffin block were stained and examined under a microscope (Olympus CX41, Tokyo, Japan).

*Anti-osteopontin* (*GmbH, Aachen, Germany*, antibodies, ABIN2774904*)*, *anti-MMP2* and *anti-MMP9 (Santa Cruz, Biotechnology, C-20):SC-6840)* were used for immunohistochemical staining. After dewaxing and microwaving in a 10 mmol citrate acid buffer (pH 6) at 95 °C for 10 minutes, the sections were cooled and then washed twice in PBS for 5 minutes. They were subsequently incubated overnight with primary antibodies (diluted 1:100) at 4 °C in a humid chamber. Slides were washed 3 times in PBS for 5 min and incubated with the secondary antibodies.

We used *goat anti-rabbit IgG secondary antibody* for OPN, MMP2, and MMP9. Slides were developed with 3,3’-diaminobenzidine (DAB) and counter-stained with hematoxylin.

## 3. Results and Discussion

### 3.1. Structural Analysis

The chemical characterization is based on the quantitative and qualitative chemical analysis of the samples taken from the ingots obtained. The chemical composition of the alloys obtained in the vacuum arc remelting furnace is presented in Table 1.

**Figure 2:**
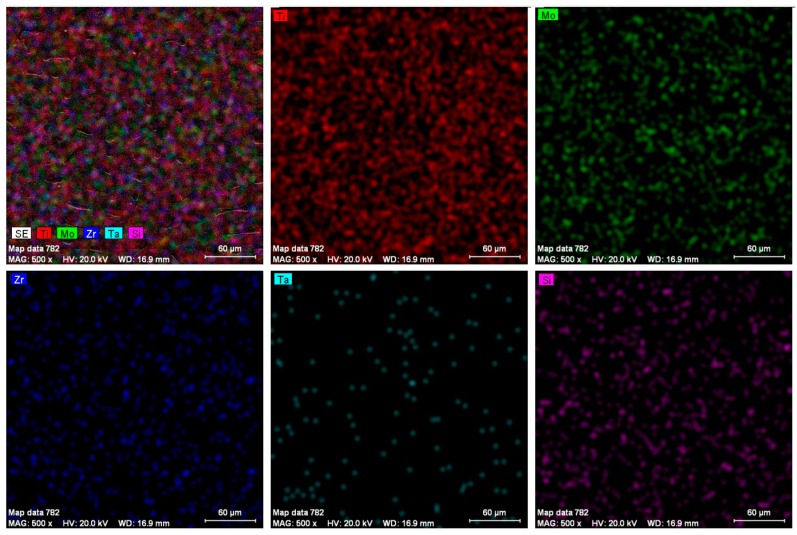
EDS elemental map analysis on specific alloy.

The chemical composition was measured for 10 different points of the samples. By analyzing the chemical composition, the constituent elements of the alloys were determined: titanium, molybdenum, tantalum, zirconium and silicon. The alloys were homogeneous, without metal inclusions. The energy-dispersive spectroscopy (EDS) elemental mapping results of the elements found in the alloys can be found in Figure 2.

**Figure 3:**
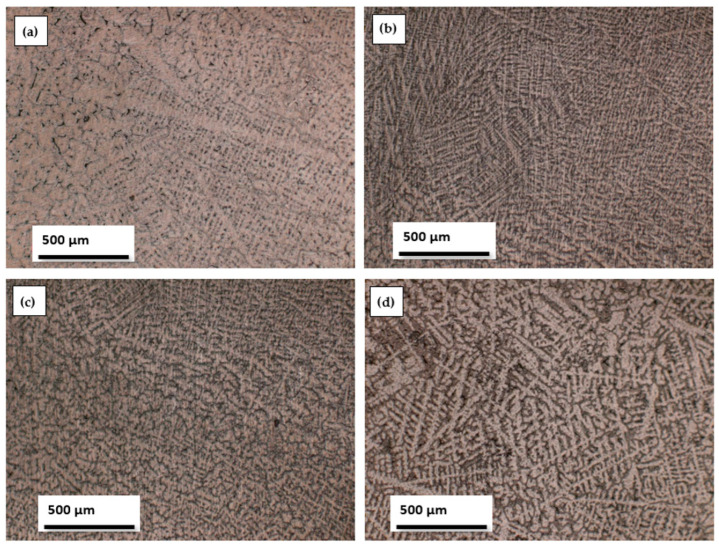
Images obtained using optical microscope: (**a**) Ti25Mo7Zr15Ta, (**b**) Ti25Mo7Zr15Ta0.5Si, (**c**) Ti25Mo7Zr15Ta0.75Si, (**d**) Ti25Mo7Zr15Ta1Si.

We conducted optical microscopy analysis on metallographically prepared samples, which were sanded with SiC metallographic paper (grits 800, 1000, 1200, or 2000) in an aqueous medium and polished with a 3 µm diamond suspension, until a mirror surface was obtained for samples with dimensions of 15.00 × 10.00 × 5.00 mm. To visualize the structure under an optical microscope, the surface of the samples was attacked with a special reagent. Figure 3 shows the structural analysis of the alloys obtained with an optical microscope.

From Figure 3, it can be observed that all alloys show a dendritic structure. One feature of the microstructure of various titanium alloys is the dendritic structure. These alloys have the ability to generate dendritic patterns.

The mechanical characteristics of titanium alloys, particularly their resistance to fatigue, can be influenced by their dendritic structure. In order to improve fatigue resistance and overall mechanical strength, titanium alloys should generally have a fine and homogenous dendritic structure [45,46].

However, additional characteristics, like the cooling rate and alloy composition, may also have an impact on the dendritic structure. A dendritic structure that is finer can be produced by rapid cooling, even though a dendritic structure that is coarser can be produced by faster cooling. Moreover, the dendritic structure and its resulting properties can be impacted via specific alloying elements [47,48].

When creating and choosing titanium alloys for use in medical implants and other applications where mechanical strength and durability are crucial, it is vital to take into account the dendritic structure in general.

The addition of beta-stabilizing elements can significantly enhance the properties of titanium alloys, making them more suitable for a wide range of applications. In our case, the beta-stabilizing elements of the introduced titanium alloys, such as Mo, Ta and Si, influenced the structure toward a dendritic structure.

**Figure 4:**
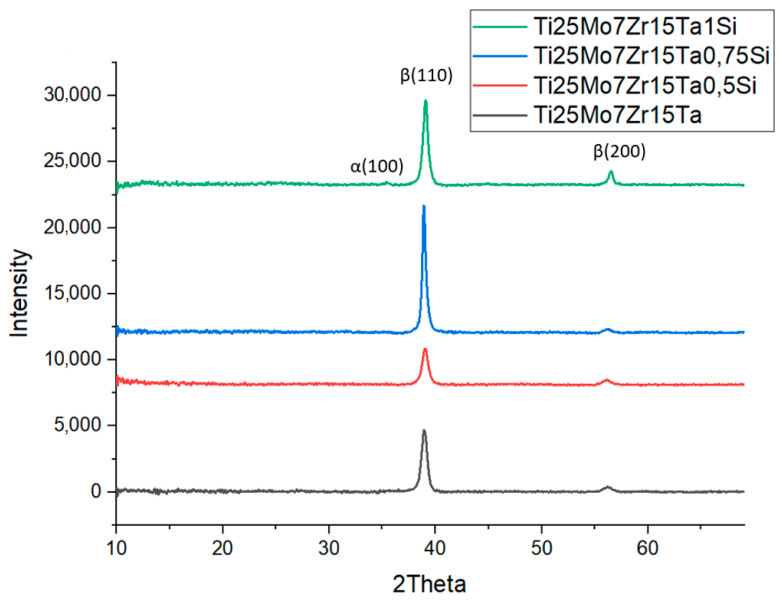
Diffraction patterns of the alloys.

Figure 4 illustrates the X-ray diffraction patterns of the studied Ti25Mo7Zr15Ta*x*Si (*x* = 0, 0.5, 0.75, 1 wt.%) alloys. XRD may be used to determine the various crystal phases that are present in titanium alloys. The alpha phase and the beta phase, the two main crystal phases of titanium alloys, each have a unique crystal structure. The structure of the studied alloys shows a majority beta-type structure, which was also confirmed via optical microscopy.

The XRD diffractograms highlight that the alloys exhibit one compound: β-Ti (reference code: 01-089-4913). The main characteristics for the Ti alloys obtained (Table 2) are as follows: crystal system: cubic, a (Å): 3.2830, b (Å): 3.2830, c (Å): 3.2830, alpha (°): 90.00, beta (°): 90.00, gamma (°): 90.00, calculated density (g/cm^3^): 4.49, the volume of the cell (10^6^ pm^3^): 35.38, Z: 2.00 and RIR: 9.61.

### 3.2. Mechanical Properties

To assess the mechanical qualities of the materials, particularly titanium alloys, indentation tests are frequently performed. In these tests, a controlled load is applied, while a hard indenter, such as a diamond tip, is driven into the material’s surface. Measurements of hardness, elastic modulus and plastic deformation behavior can all be made using the resulting indentation.

Indentation testing on titanium alloys can reveal important details about the material’s mechanical properties at micro- and nanoscales. For instance, nanoindentation studies can be used to gauge the elastic modulus and hardness of distinct alloy phases, like the alpha and beta phases. These measurements can shed light on how alloying components and manufacturing parameters affect the material’s mechanical properties.

Indentation tests can be performed to examine the deformation behavior of titanium alloys at micro- and nano-scales in addition to analyzing their bulk mechanical properties. Researchers can learn details about the material’s plastic deformation behavior, including the size and distribution of deformation zones and the level of strain hardening, by examining the shape and size of the indentation. For some applications, this knowledge can be used to enhance the design and processing of titanium alloys.

Indentation tests are generally a good method for assessing the mechanical qualities of titanium alloys. At micro- and nanoscales, they can offer insightful data about the hardness, elastic modulus and plastic deformation behavior of a material, which can be used to improve the alloy’s characteristics and functionality for a variety of applications.

**Figure 5:**
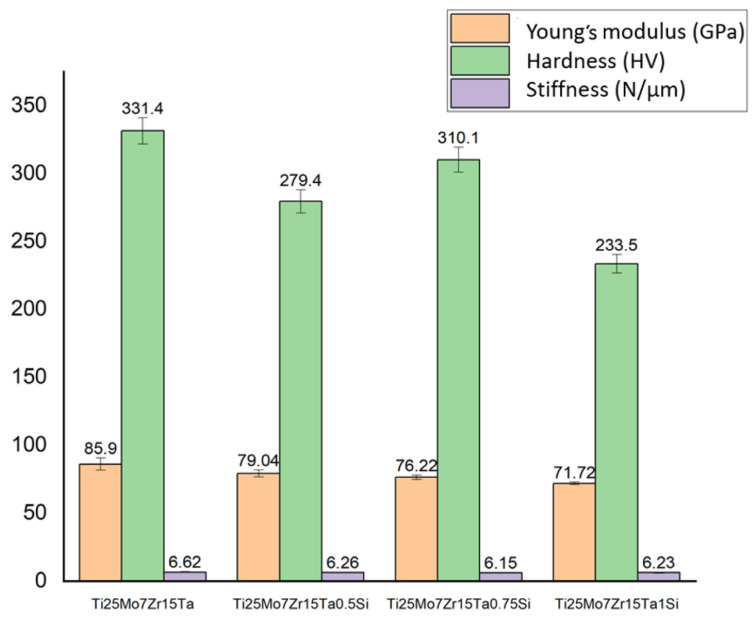
The mechanical properties of the alloys obtained via indentation.

Figure 5 displays the average values for 10 tests (±standard deviation) obtained from the indentation measurements of the Ti-Mo-Zr-Ta-Si alloys. Hardness measurements were conducted, indicating a range of 233.5 to 331.4 HV, with hardness decreasing as the percentage of silicon increased. The indentation method was used to determine the modulus of elasticity, which fell between 71.72 and 85.9 GPa. In general, the inclusion of small quantities of silicon (0.1% to 1.0%) in titanium alloys has a beneficial impact on their mechanical characteristics by reducing the modulus of elasticity.

In recent years, the continuous search for titanium alloys with improved mechanical and biological properties has yielded a variety of novel compositions. A crucial aspect of these newly developed alloys is the optimization of their Young’s modulus to closely resemble that of human cortical bone (15–30 GPa), which is crucial for orthopedic applications to minimize the stress shielding effect.

For our Ti25Mo7Zr15Ta1Si alloy, we observed that Young’s modulus is higher than that of cortical bone (71.72 GPa). However, a remarkable trend was noted: with an increase in Si content, there was a noticeable decrease in Young’s modulus. This offers the possibility of tuning the mechanical properties of the alloy by varying the Si content, a significant advantage for specific biomedical applications.

Compared with a classic titanium alloy commonly used in orthopedic applications, Ti-6Al-7Nb has the highest Young’s Modulus (105 GPa). Its high modulus, however, can result in stress shielding effects when used as an implant material.

Another alloy, Ti-19Nb-14Zr stands out as the alloy with the lowest modulus, coming in at just 14 GPa. This makes it considerably more elastic and closer to the modulus of human bone, potentially reducing stress shielding effects.

Our Ti25Mo7Zr15TaxSi alloy, depending on the amount of Ta, has an average modulus of 79.22 GPa. This places it as mid-range in comparison to the other alloys. Notably, it matches the modulus of Ti-12Mo-5Zr, suggesting similarities in their mechanical properties (64 GPa). What is important, however, is that by varying the Si content in the system, there is potential to tune its Young’s modulus, a unique capability not necessarily present in these other compositions.

The addition of silicon is seen in alloys like Ti-15Mo-1Si (43 GPa) and Ti-20Mo-1Si (26 GPa) generally seems to reduce the modulus. The lowest is Ti-15Mo-0.5Si at 20 GPa, which is quite close to the modulus of bone.

### 3.3. Interpretation of Results, Radiographs and Micrographs

**Figure 6:**
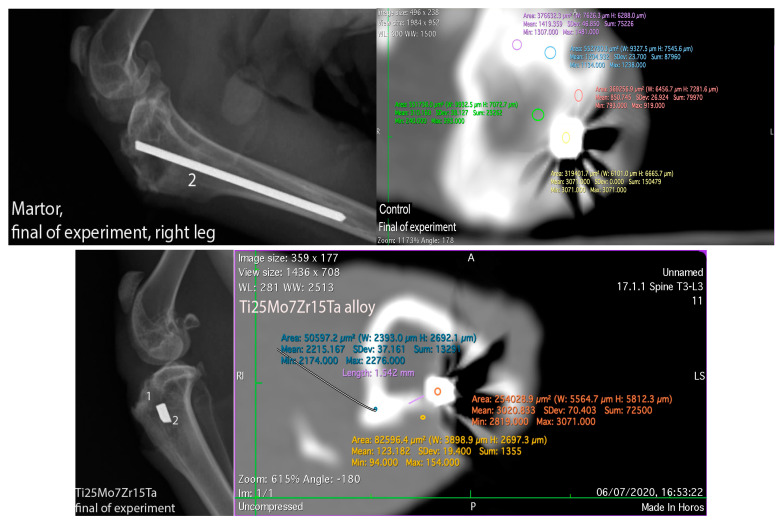

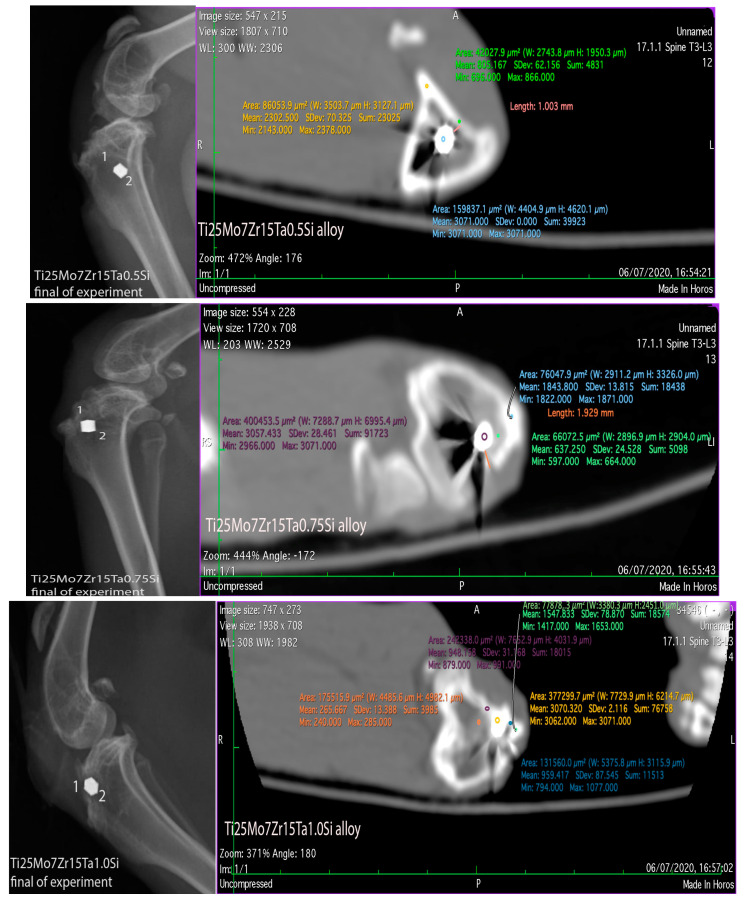
X-Ray and CT-scan at control and experimental rabbits at the final stage of experiment, 1—surgical breach, 2—alloy.

The primary objective of the X-ray analysis was to determine the position of the alloys in both the control and experimental rabbits, as well as to detect any relevant reactions in the surrounding tissues to the alloy surface. Throughout the 60-day experimentation period, no abnormal radiological events were observed (Figure 6).

*CT Analysis:* We utilized a high-resolution CT system (Siemens Somatom Balance, Erlangen, Germany) with a 20 µm/slice scanning resolution to assess peri-implant tissue density. The resulting images were processed into both 3D and 2D formats using Image Software (Version 2.21 Synego viewer). Density measurements were made using Hounsfield Units (HU), revealing that the alloy structure exhibited higher density than surrounding bones and tissues, leading to the creation of artifacts.

**Figure 7:**
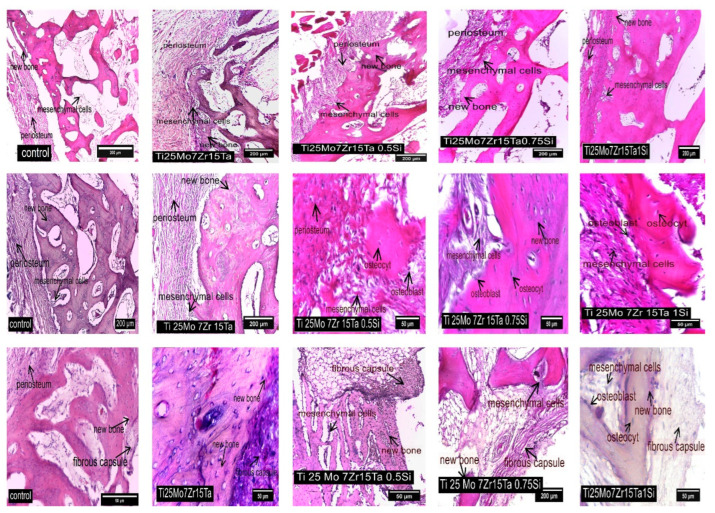
Osteogenesis Aspects in Control and Experimental Rabbits: The first row illustrates periosteum activity near the implant in both control and experimental rabbits using HE staining. The middle row showcases osteogenesis in the proximity of the periosteum, depicting aspects of new bone formation in both control and experimental rabbits through HE staining. The bottom row provides insights into the structure of the fibrous capsule and the development of new bone in both control and experimental rabbits, as observed in HE staining.

Regarding the radiodensity of the surrounding tissues of the peri-implant [49,50], the newly regenerative tissue in the surgical gap measured about 793 UH (the compact bone measured about 1500 UH). For the Ti25Mo7Zn15Ta1.0Si alloy, radiopacity ranged from 794 to 991 Hounsfield Units (HU) compared to the compact bone, which measured about 1417–1653 HU. The Ti25Mo7Zr15Ta0.5Si alloy exhibited radiopacity in the range of 696–866 HU compared to the tibial compacta, which measured about 2143–2378 HU. The Ti25Mo7Zr15Ta0.75Si alloy showed radiopacity between 597 and 664 HU, while the tibial compacta measured between 1822 and 1871 HU (see Figure 6). In contrast, the Ti25Mo7Zr15Ta alloy displayed a lower peri-implant radiopacity, measuring up to 150 HU, compared to the bone compacta, which measured between 2174 and 2276 HU (see Figure 7). The radiopacity observed in the newly formed tissues around the implants correlated with the presence of fibrous tissues, particularly in the case of intramembranous ossification. For Ti25Mo7Zr15Ta, this correlation was reinforced by the presence of cartilage islands and ossification punctures, as confirmed by histological analyses [16,23,51].

In the control rabbits, mesenchymal stem cells were located in the inner layer of the periosteum and differentiated into osteoblasts, responsible for synthesizing preossein and collagen fibers (see Figure 7). Preossein underwent mineralization, resulting in ossein. Various bone structures, including osteoids, osteons, and bone trabeculae, were observed. Additionally, within cartilaginous islands, osteocytes were found in the lacunae of mineralized ossein (see Figure 7).

A passive layer, ranging from 3 to 10 nm, covers the titanium alloy and has the capacity to attract calcium and phosphate ions, leading to the binding of specific proteins such as collagen type I and osteocalcin to osteoblasts. This aspect was also observed in our experiment [52,53,54,55,56].

For all implants, stem cells are present in the cambium area, which differentiate into osteoprogenitor cells that later become osteoblasts involved in osteoid synthesis [21]. Adjacent to the periosteum, trabecular bone tissue forms, extending into compact bone tissue (see Figure 7, middle row). Osteoblasts are present along the peri-trabeculae, and osteoprogenitor stem cells are found in the bone areolae, between the peri-implant fibrous capsule and the newly formed bone tissue [57,58]. 

In the case of the Ti25Mo7Zr15Ta alloy, the proliferation of osteoprogenitor cells and osteoid formation can be observed in cartilage islands. The newly formed bone tissue consists mainly of fibro-cartilaginous tissue, including rare islands of mineralization. The inner layer of the periosteum continues with islands of mineralization of the newly formed bone tissue (Figure 7). In the case of the Ti25Mo7Zr15Ta0.5Si alloy, the newly formed tissue surrounding the implant is predominantly cartilaginous and includes mineralized islands and bone lamellae (Figure 7).

**Figure 8:**
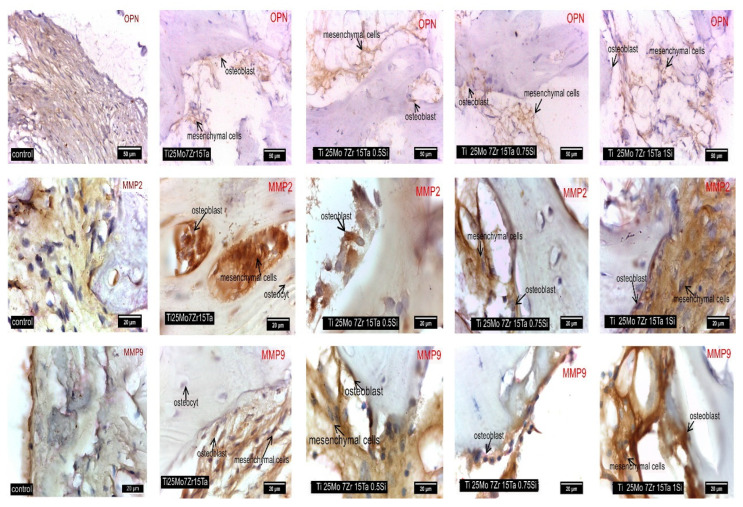
Staining for OPN, MMP2, and MMP9 in Control and Experimental Rabbits Near the Alloy: The first row of images displays cells positive for Osteopontin (OPN) using IHC staining. The second row shows cells positive for Metalloproteinase 2 (MMP2) using IHC staining. The third row presents cells positive for Metalloproteinase 9 (MMP9) using IHC staining.

In the case of the Ti25Mo7Zr17Ta0.75Si alloy, the peri-implant fibrous capsule is thinner and denser, and the newly formed bone tissue consists of an alternation of cartilage islands with mineralized areas and the formation of bone lamellae (Figure 8). The peri-implant fibrous capsule of Ti25Mo7Zr15Ta1.0Si continues with newly formed bone tissue rich in osteocytes and cartilage islands where osteoblasts are identified. In the inner layer of the periosteum, rich osteogenic activity is identified, being intensely vascularized and rich in mesenchymal cells, osteoblasts and osteocytes (Figure 8).

OPN is produced via embryonic stroma, macrophages, fibroblast cells or bone cells, such as osteoblasts, in different stages of differentiation, and osteocytes are involved in the wound-healing process of bones, contributing to osteoclast genesis and activity [59,60,61,62,63,64]. It contains arginine–glycine–aspartate (RGD) and has a role in promoting cell adhesion by inducing MMP-9 or MMP-2 secretion and regulation in various cells [65].

In this study, the overexpression of OPN in the bone cells is an indicator of progressive osteogenesis. In the control rabbit, OPN was observed, and it exhibited overexpression in the bone interface area in the experimental rabbits (see Figure 8) [16,23,59]

In the current study, high MMP-9 activity was highlighted in osteoprogenitor cells and osteoblasts. Our results suggest that MMP-9 is the downstream molecular signal of OPN, thus contributing to cell migration [61,66]. MMPs belong to the zinc-dependent endopeptidases family and represent the main class of enzymes involved in the degradation and resorption of all extracellular matrix compounds. MMP-2 presence is required in bone embryonic development, tissue repair and tumor genesis [67]; however, MMP-9 plays an important role in bone remodeling as a result of osteoclastic activity [68,69,70]. In our study, the overexpression of MMP9 and MMP2 in the periosteal and peri-implant cells was registered in all control and experimental rabbits (Figure 8), showing a normal process of bone reconstruction.

## 4. Conclusions

In recent years, the medical field has seen a surge in the use of metallic biomaterials, especially for orthopedic and dental implants. However, their use is not just limited to these fields; new applications are continually being discovered. Each of these materials is carefully assessed and undergoes stringent testing to ensure they meet the required safety and efficacy standards for medical applications.

While traditional alloys like C.P.-Ti, Ti6Al4V and Ti6Al7Nb have played a significant role in medical implantology, the research world has constantly been looking for improved materials with better mechanical and biocompatible properties. Newly developed Ti-alloys, like the one presented in this work, offer potential enhancements over their predecessors.

Our study focuses on the development of a new Ti25Mo7Zr15TaxSi (*x* = 0, 0.5, 0.75, 1 wt.%) alloy, which was synthesized in a vacuum electric arc furnace. This alloy was rigorously characterized in terms of its structure, mechanical properties and biocompatibility.

Optical microscopy highlighted its dendritic structure, which is specific to titanium alloys due to the beta-stabilizing elements introduced into the composition of the alloys. This was also evidenced via X-ray diffraction, with the majority being the beta phase.

The results of the main mechanical properties measured by indentation were visibly influenced by the addition of silicon; as the percentage of silicon increased, both the hardness and the modulus of elasticity decreased, exhibiting a beneficial role. 

Through histological and immunohistochemical investigations (OPN, MMP2 and MMP9), the development of peri-implant bone regeneration was followed. Thus, both in the control and experimental rabbits, at the implantation gap, intense proliferative activity was observed in the internal layer of the periosteum and on the periphery of the peri-implant fibrous capsule. The presence of cells positive for OPN, MMP-2, and MMP-9 indicates the proliferation and migration of cells that enhance osseointegration and extracellular matrix production. These molecules facilitate cell adhesion, leading to callus remodeling and the redirection of trabeculae in newly formed bone along the force lines of the osteon system, ultimately promoting the integration of the alloy into the bone. 

Due to their biocompatibility and strength, titanium alloys have generally shown to be a dependable and successful material for medical implants, making them the perfect option for surgical implants that must stay in the body for extended periods of time.

The Ti25Mo7Zr15TaxSi alloy system exhibits a lower modulus of elasticity compared to classic alloys, such as C.P.-Ti, Ti6Al4V and Ti6Al7Nb, and offers superior biocompatibility. Titanium alloys, owing to their biocompatibility and strength, have stood the test of time as dependable materials for medical implants. Their reliability becomes even more crucial for surgical implants that are designed to remain within the body for prolonged durations. The newly developed Ti-Mo-Zr-Ta-Si system, based on our findings, possesses the potential for medical applications, most notably within the orthopedic sphere, without any risk of bodily rejection.

## Figures and Tables

**Table 1:** Chemical composition for alloys obtained.

Sample		Ti25Mo7Zr15Ta	Ti25Mo7Zr15Ta0.5Si	Ti25Mo7Zr15Ta0.75Si	Ti25Mo7Zr15Ta1Si
Average chemical composition	Ti (wt.%)	53.40 ± 0.60	52.60 ± 0.8	52.49 ± 0.70	54.00 ± 0.60
	Mo (wt.%)	24.90 ± 0.30	24.70 ± 0.20	24.80 ± 0.50	23.60 ± 0.40
	Zr (wt.%)	6.90 ± 0.10	7.10 ± 0.20	6.90 ± 0.10	6.80 ± 0.30
	Ta (wt.%)	14.80 ± 0.40	15.20 ± 0.30	15.10 ± 0.20	14.70 ± 0.50
	Si (wt.%)	-	0.40 ± 0.01	0.71 ± 0.05	0.90 ± 0.09

**Table 2:** The main characteristics of XRD diffractograms.

Crystal System	a (Å)	b (Å)	c (Å)	Alpha (°)	Beta (°)	Gamma (°)	Calculated Density (g/cm^3^)	Volume of the Cell (10^6^ pm^3^)	Z	RIR
Cubic	3.2830	3.2830	3.2830	90.00	90.00	90.00	4.49	35.38	2.00	9.61

## Data Availability

Data are contained within the article.
